# Re-emergence of Lloviu virus in *Miniopterus schreibersii* bats, Hungary, 2016

**DOI:** 10.1038/s41426-018-0067-4

**Published:** 2018-04-18

**Authors:** Gábor Kemenesi, Kornélia Kurucz, Bianka Dallos, Brigitta Zana, Fanni Földes, Sándor Boldogh, Tamás Görföl, Miles W Carroll, Ferenc Jakab

**Affiliations:** 1 Szentágothai Research Centre, Pécs, H-7624 Hungary; 2 Aggtelek National Park Directorate, Jósvafő, H-3758 Hungary; 3 Department of Zoology, Hungarian Natural History Museum, Budapest, H-1083 Hungary; 4 Public Health England, Porton Down, Salisbury, SP4 0JG UK

Members of the virus family *Filoviridae*, such as ebola viruses and marburgviruses, cause hemorrhagic fevers associated with high mortality and are, therefore, of major public health importance worldwide^1^. The 2013–2016 West African Ebola virus (EBOV) disease outbreak further illustrated the epidemic potential and devastating effects associated with the members of *Filoviridae*. Bats have been identified as reservoirs for marburgviruses and are suspected to be involved in the transmission of ebola viruses to humans^2,3^. Since the discovery of Lloviu virus (LLOV) in *Miniopterus schreibersii* samples from Spain in 2002, European bats have also been considered as a possible host for filoviruses^4^. Subsequently, there have been no additional reports of LLOV detection or isolation, resulting in a paucity of knowledge on the biology and pathology of the virus. Moreover, no specific diagnostic test has been described, which further hampers surveillance studies^5^. Furthermore, the identification of genetically diverse filoviruses discovered in Chinese bat colonies has further highlighted the broad geographical distribution of filoviruses and their unexpected genetic diversity^6,7^.

In 2013, a mass-mortality incident occurred in a *M. schreibersii* bat colony, causing the death of ~500 individuals in Northeast Hungary (Bükk mountain). After investigating the scene, only aged carcasses could be collected, which prevented virological investigation. Blood coagulate was detected on the nose of several individuals, referring to possible respiratory hemorrhages. Other known bat colonies in the area were also checked and were found to be unaffected at that time. Three years later, in 2016, five Schreiber’s bat individuals were reported with the gross pathologic symptom of respiratory bleeding (Supplementary material) in Northeast Hungary ~50 km from the site of the 2013 incident. Interestingly, all five individuals were located close to the entrance of the mine cavern system. Seven other *M. schreibersii* bats were hibernating deeper in the mine and were separated from the sick individuals, which was the same location of the bats observed during annual winter counting (in the summer, the maternity colony generally consists of 5–6000 *M. schreibersii* individuals). The bats inside the mine cavern system were unaffected at the time of sampling and were frequently monitored during 2017. An additional mortality event was detected in December 2017 that affected 25 individuals roosting separately. Unfortunately, the decaying condition of the carcasses did not permit virological examination; thus, a connection to LLOV infection cannot be made. On the basis of a previous visit to the cave site in July, we hypothesize that this latest *M. schreibersii* mortality event occurred between July and December 2017.

Fresh carcasses from 2016 were collected and transported on dry ice to the biosafety level 4 (BSL-4) suit laboratory at the Szentágothai Research Centre, University of Pécs, for further examination. Briefly, after dissection, tissue samples were homogenized, and nucleic acid was extracted using a QIAamp Viral RNA Mini Kit (QAIGEN) according to the manufacturers’ recommendations. Nested RT-PCR specific for the RNA-dependent RNA polymerase gene was used to identify filovirus RNA as described previously^6^. The RT-PCR amplicon was purified from agarose gel, and then bi-directionally sequenced on an ABI Prism 310 Genetic Analyzer. On the basis of the preliminary sequence data for the Hungarian LLOV, a specific TaqMan probe-based real-time RT-PCR assay was designed (LLOV-Fw-scr: 5′-ACTGACATTGGAAAACCGAGG-3′; LLOV-Rev-scr: 5′-CCTCAATCCCTCCAAGATGTC-3′; LLOV-Prob-scr: 5′-FAM-CTGTATGAACTTGGACCCTCGGGC-ZEN-3′). The One-Step RT-PCR (QIAGEN) conditions were as follows: 50 °C for 30 min, 95 °C for 15 min followed by 50 cycles of 94 °C for 30 s, 51 °C for 30 s and 72 °C for 1 min. To the best of our knowledge, this is the first published LLOV-specific diagnostic assay used on wildlife samples. To further support the presence of LLOV in our samples, we designed two additional nested primer sets specific to the nucleoprotein gene. The nucleoprotein-specific oligonucleotide sequences and nested RT-PCR reaction conditions are summarized in the Supplementary material. No positive control was available in our laboratory during the experiments, excluding any false positive PCR results from nucleic-acid contamination.

Filovirus RNA was detected in one of the five lung samples tested by RT-PCR; that sample was the most well preserved. It is likely that the poor condition of bat carcasses and related degradation of viral nucleic acids explains the lack of signal in the other four lung samples examined. Since the causality between the known mortality events of bats and LLOV infection has not been shown, any other cause of death is also possible. A Basic Local Alignment Tool (BLAST) search (www.ncbi.nlm.nih.gov) and further phylogenetic analyses showed that the Hungarian virus (LLOV/Mad/2016/Hungary; accession number: MF996369) was most closely related to LLOV isolate MS-Liver-86/2003 (JF828358) with 98% nucleic acid homology (Fig. 1). This partial RNA-dependent RNA-polymerase sequence does not allow us to draw concrete conclusions regarding the evolution of LLOV in the past several years. The partial sequence does indicate the presence or re-emergence of a closely related variant to the Spanish LLOV in Hungary after more than a decade. As a result of nucleoprotein-specific nested RT-PCR reactions, we were able to amplify two additional fragments of the genome representing partial sequences of 747 and 326 nucleotides from the nucleoprotein gene (Accession numbers: MG888042 and MG888043). These fragments were almost identical to LLOV isolate MS-Liver-86/2003 from Spain with 99% nucleotide homology. Phylogenetic analysis on the 747-bp gene fragment further supported the close genetic and evolutionary relationship of the Hungarian and Spanish LLOV isolates (Fig. 2). Using our real-time RT-PCR approach, we investigated the tissue tropism of the virus by testing similar amounts of tissue from several organs derived from the LLOV positive individual. Lung and spleen samples were positive with similar Ct values (35.56 and 35.30, respectively), whereas the liver, kidney, brain and a tick removed from the carcass were negative for LLOV-specific nucleic acids. The exact tissue tropism of the virus is not totally clear, despite the similarly between our results and other studies that primarily detected LLOV and diverse filovirus RNA in lung samples from Spain and China, respectively^4,7^. Interestingly, no gross pathology of respiratory bleeding was reported prior to our study, although symptoms consistent with viral pneumonia were described in the Spanish samples^4^.

Virus isolation was carried out under BSL-4 suit laboratory conditions using an African green monkey kidney cell line (Vero E6; ATCC® CRL-1586). Briefly, Vero E6 cells were prepared on a 24-well cell culture plate using Eagle’s minimum essential medium (MEM) with 10% fetal bovine serum (FBS) and antibiotics (streptomycin and ampicillin) as a growth medium. Plates with 80–90% confluent cell monolayers were washed with phosphate buffered saline, and then 200 μL from all examined tissues (brain, lung, liver, kidney and spleen) homogenized in phosphate buffered saline solution was added. The inoculated plates were incubated at 37 °C in a CO_2_ incubator. Ten days post infection, freeze/thaw cycles were performed three times, and then additional blind passages were conducted two more times. No cytopathic effect was visible on the first inoculation or on additional blind passages. Specific viral RNA was not detected from any of the infected cell media. Interestingly, there was no indication of in vitro isolation procedures for LLOV in the literature^4^. In vitro experiments of LLOV from positive Spanish samples were discussed by Burk et al.^5^ but unfortunately, all sample materials connected with the Spanish mortality events were depleted, prohibiting further experimentation or analysis. Moreover, similar to our results, studies in China reported in vitro isolation procedures in Vero E6 cell culture without successful isolation of any detected viruses^6,7^.

Although the human pathogenic potential of the virus is still unknown, the innate immune inhibitory function of LLOV-derived VP35 and VP24 proteins in human and bat cells has been reported^8^. Moreover, other studies involving recombinant LLOV proteins reported EBOV reminiscent receptor usage^9,10^. To assess the potential role of the virus in human or primate infections, further biological, molecular and in vitro investigations are needed.

More than a decade after the mass mortality of *M. schreibersii* bats in Spain (Cueva del Lloviu), LLOV has re-emerged in Europe, and we report its presence within the same bat species in Hungary. This incidence raises the question of whether it was a second introduction of LLOV in Europe or an unnoticed silent circulation proceeding among European bats. Our finding indicates a significant range expansion of the virus from the Iberian Peninsula of Spain, its currently only known geographic range. Due to the possible connection between LLOV infection and the mortality of *M. schreibersii*, it seems that this species is not the reservoir animal of LLOV. To clarify the exact source (i.e., other bat species, insects or additional animal species living in the same habitat) and geographic range of LLOV, further investigations involving bat-connected ecological niches are needed without unnecessary disturbance. The potential ecological ramifications of the negative effects on local bat populations have been reported for White Nose Syndrome in NorthvAmerica^11^. *M. schreibersii* and closely related *Miniopterus* species are widely distributed across Eurasia and have been known to roost in huge colonies of up to several thousand individuals^12^. These bats represent a significant concern regarding conservation biology since they represent an important ecological resource and an integral part of European biodiversity. It seems that LLOV may cause severe disease in one species of bat, resulting in mass mortalities in colonies. Therefore, increased attention from the scientific community and conservation biologists is required. Although any direct causality between mortality events in *M. schreibersii* and LLOV infection has not been shown, our findings of LLOV range expansion demonstrate the need for further investigation.

**Fig. 1:**
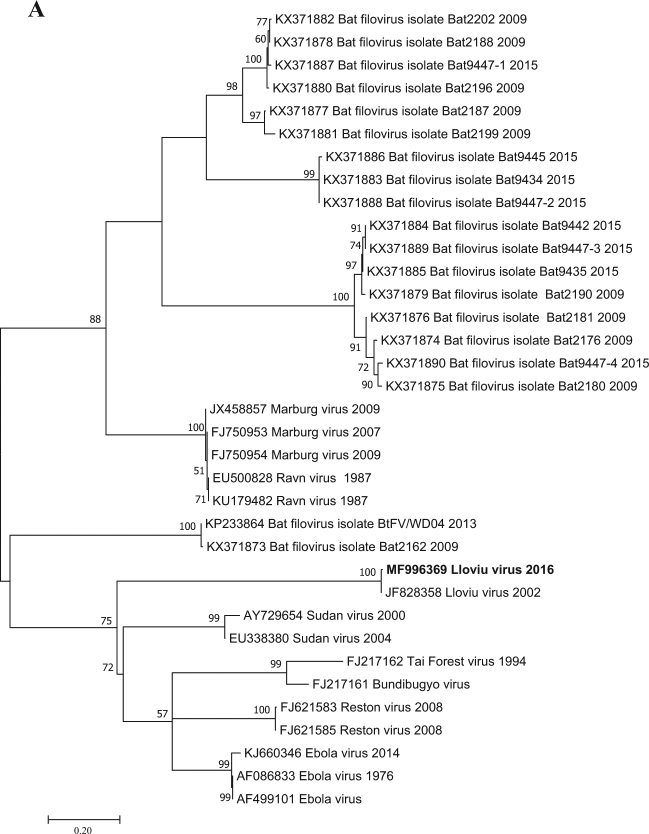
Phylogenetic analysis was performed based on a 346-nt partial sequence of the RNA-dependent RNA polymerase gene with the Maximum Likelihood method based on the Tamura 3-parameter model (+G+I). For the maximum-likelihood tree, the best fit nucleotide substitution model was selected based on the Bayesian information criterion as implemented in MEGA7 software^13^. Bootstrap replications were 1000. The sequence in this study is indicated with bold letters. Branch supports are represented by bootstrap values. Bootstrap values less than 50 are not shown. Branch lengths are measured in the number of substitutions per site.

**Fig. 2:**
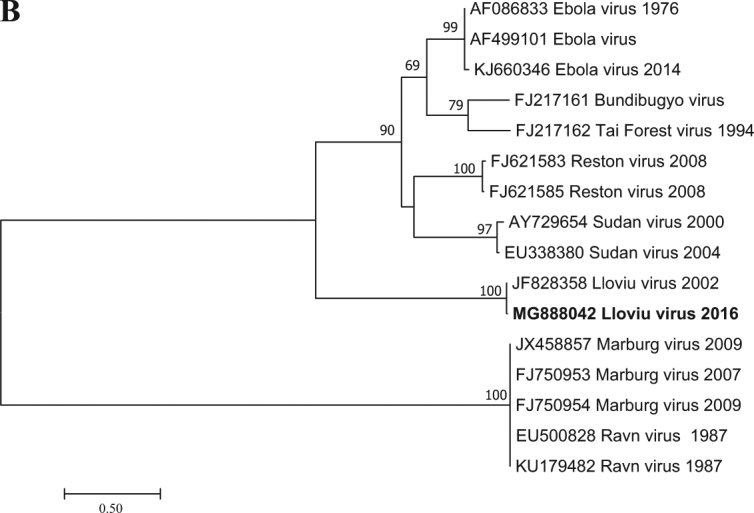
Phylogenetic tree analysis was performed based on a 717-nt partial sequence of the nucleoprotein gene with the Maximum Likelihood method based on the Tamura 3-parameter model (+G+I) in MEGA7 software^13^. Bootstrap replications were 1000. The sequence of this study is indicated with bold letters. Branch supports are represented by bootstrap values. Bootstrap values <50 are not shown. Branch lengths are measured in the number of substitutions per site

## Electronic supplementary material

supplemental Table and Figure

## References

[1] Feldmann H, Klenk HD (1996). Marburg and Ebola viruses. Adv. Virus Res..

[2] Coltart CEM, Lindsey B, Ghinai I, Johnson AM, Heymann DL (2017). The Ebola outbreak, 2013–2016: old lessons for new epidemics. Philos. Trans. R. Soc. Lond. B: Biol. Sci..

[3] Amman, B. R., Swanepoel, R., Nichol, S. T. & Towner, J. S. in: *Marburg- and Ebolaviruses. Current Topics in Microbiology and Immunology* Vol 411 (eds Mühlberger E., Hensley L. & Towner J.) (Springer, Cham, 2017) 10.1007/82_2017_10 28710694

[4] Negredo A (2011). Discovery of an ebolavirus-like filovirus in Europe. PLoS Pathog..

[5] Burk R (2016). Neglected filoviruses. FEMS Microbiol. Rev..

[6] He B (2015). Filovirus RNA in fruit bats, China. Emerg. Infect. Dis..

[7] Yang XL (2017). Genetically diverse filoviruses in rousettus and eonycteris spp. bats, China, 2009 and 2015. Emerg. Infect. Dis..

[8] Feagins AR, Basler CF (2015). Lloviu virus VP24 and VP35 proteins function as innate immune antagonists in human and bat cells. Virology.

[9] Maruyama J (2014). Characterization of the envelope glycoprotein of a novel filovirus, Lloviu virus. J. Virol..

[10] Ng, M.et al. Cell entry by a novel European filovirus requires host endosomal cysteine proteases and Niemann-Pick C1. Virology 637–646 (2014). 10.1016/j.virol.2014.08.019 PMC4252868 25310500

[11] Frick WF (2010). An emerging disease causes regional population collapse of a common North American bat species. Science.

[12] Appleton BR, McKenzie JA, Christidis L (2004). Molecular systematics and biogeography of the bent-wing bat complex Miniopterus schreibersii (Kuhl, 1817) (Chiroptera: Vespertilionidae). Mol. Phylogenet. Evol..

[13] Kumar S, Stecher G, Tamura K (2016). MEGA7: molecular evolutionary genetics analysis version 7.0 for bigger datasets. Mol. Biol. Evol..

