# Supplementary material for: Re-emergence of Lloviu virus in Miniopterus schreibersii bats, Hungary, 2016
Source: Emerg Microbes Infect. 2018 Apr 18;7:66. doi: 10.1038/s41426-018-0067-4 (PMC5906664; doi:10.1038/s41426-018-0067-4)
Supplement: Supplementary file 1 — supplemental Table and Figure [file 41426_2018_67_MOESM1_ESM.docx]

Nested RT-PCR oligonucleotide sequences used for amplifying nucleoprotein gene fragments of Lloviu virus Hungarian isolate are listed as **Table**. A1 and B1 indicates first round of nested RT-PCR reaction primers, whilst A2 and B2 indicates the second round primers. QIAGEN OneStep RT-PCR Kit (Qiagen, Germany) was used for first round of reactions and GoTaq® G2 DNA Polymerase Kit (Promega, USA) was used for second round of reactions. Reaction conditions were as follows for the first round of nested RT-PCRs: 50 °C 30 min, 95 °C 15 min; 40 cycles of 94 °C 30 sec, 48 °C 30 sec, 72 °C 45 sec. Conditions for second round of PCRs were: 95 °C 2 min; 40 cycles of 94 °C 30 sec, 48 °C 30 sec, 72 °C 45 sec.

| **Oligonucleotide** | **Sequence (5’->3’)** | **Polarity** | **Expected size** |
| --- | --- | --- | --- |
| LLOVNP_A1_367_F | GTA GGG AGT CGA GAC AAA TC | + | 944 bp |
| LLOVNP_A1_1311_R | CCG TAT CCC CCT ATT TTC TG | - |  |
| LLOVNP_A2_374_F | GTC GAG ACA AAT CAC TCA GA | + | 815 bp |
| LLOVNP_A2_1189_R | TTG CTA ATA TCG AGC TGG TC | - |  |
| LLOVNP_B1_1291_F | CCA GAA AAT AGG GGG ATA CG | + | 795 bp |
| LLOVNP_B1_2086_R | GGT TGT TGT TTG AGG TGA AG | - |  |
| LLOVNP_B2_1320_F | GGG ATC AAC CAA AGA GTC AT | + | 405 bp |
| LLOVNP_B2_1725_R | AAA GCT GAT TAA TGG GAG GG | - |  |

Photo of bat carcasses at the sampling site on 11 February 2016 taken by S. Boldogh. Hemorrhagic symptoms are visually detectable on all specimens. LLOV RNA was detected in the lung and spleen samples of the least degraded individual (first from the left).

**
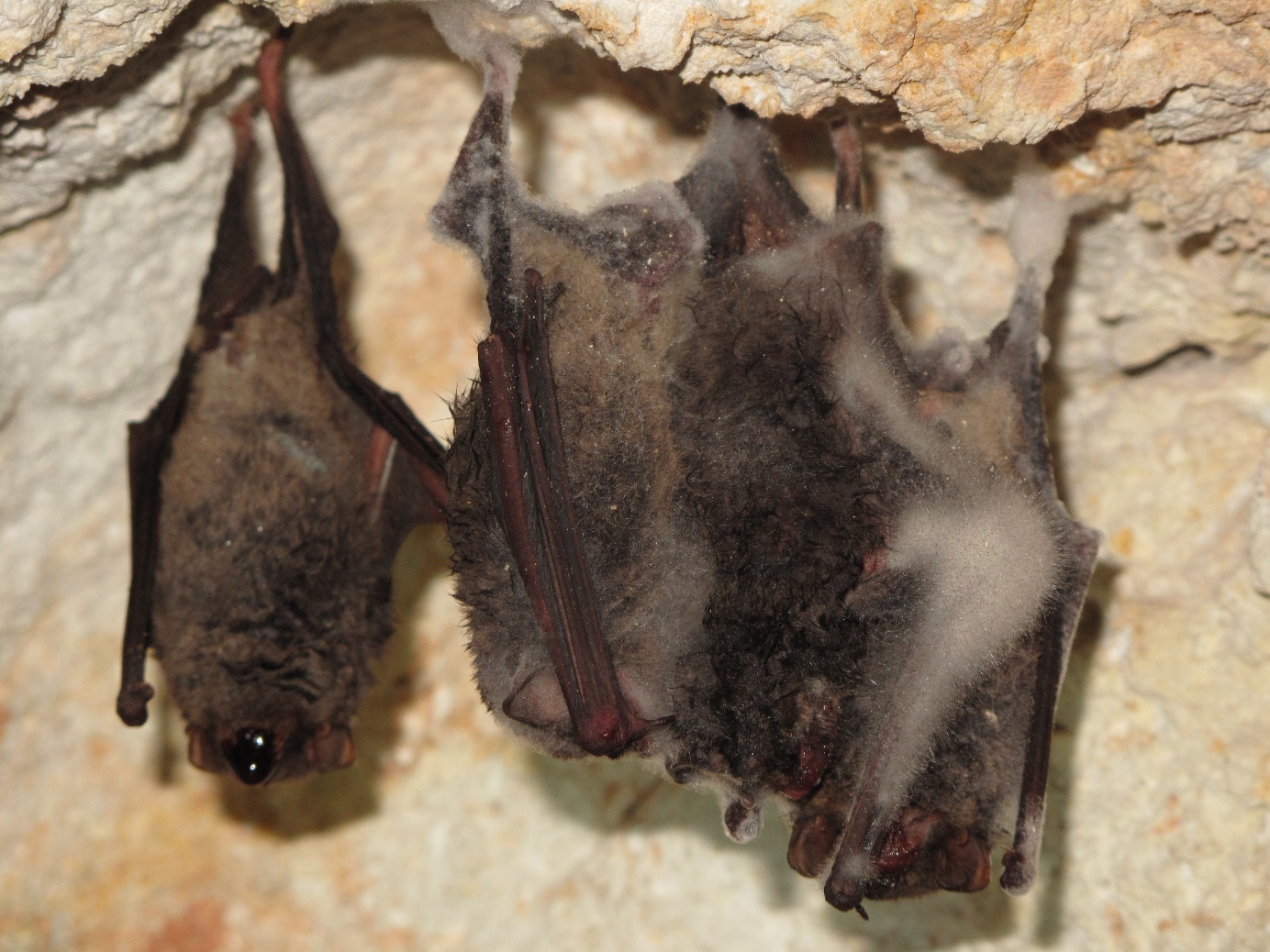
**
